# Talking across worlds: The ontological turn and communication in natural resource co-management with Indigenous communities

**DOI:** 10.1177/27539687241269336

**Published:** 2024-08-06

**Authors:** Suzanne Chew

**Affiliations:** Department of Geography, 2129 University of Calgary, Calgary, Canada Department of Geography, Geomatics and Environment and Department of Anthropology, University of Toronto Mississauga, Mississauga, ON, Canada

**Keywords:** communication, comanagement, Indigenous, ethical equivocation, ontology, onto-epistemic violence, ethical space, ethical relationality

## Abstract

The ontological turn in critical social theory provokes emerging calls for new approaches to natural resource management where Indigenous perspectives, worldview, knowledges, and values are prioritized in the stewardship of Indigenous lands. Yet, scant literature focuses on the ontological implications for communication within environmental decision-making, where Habermas’ communicative action theory, with its norms of privileging argumentation, formality, expertise, institutional authority, rationality, and language, continues to shape spaces of public participation since the communicative turn in the 1990s. Growing calls for participatory decision-making, as well as the mounting failures of scientific management approaches espoused by conventional natural resource management, have fuelled the rise of co-management since the 1980s. The emerging emphasis in co-management approaches on community collaboration and meaningful communication was strengthened with the emergence of adaptive co-management and adaptive governance in the early 2000s. Yet, the ontological turn unveils communicative tensions which continue to persist, rooted in ontological difference and onto-epistemic violence. Rethinking communication under the ontological turn in co-management with Indigenous communities, this paper reviews the literature and further proposes the idea of ethical equivocation as a communicative tool and starting point toward learning to talk across worlds.

## Introduction

Within natural resource management and environmental governance, the ontological turn has led to calls for new approaches where Indigenous perspectives, worldview, knowledges, and values are prioritized in the stewardship of Indigenous lands (see Bawaka Country Including S. Wright et al. 2016; White 2020; Blaser 2014; Watson and Huntington 2014). Alongside these calls are two emerging trends—first, the growing scholarship elaborating Indigenous futurities, brilliance, self-determination, and approaches to governance, including environmental governance (see Tynan 2021; Whyte 2017; Watts 2013; Atleo 2011; Coulthard 2017). Second, within the field of biodiversity conservation, researchers and practitioners are increasingly turning towards learning from Indigenous peoples’ land stewardship and qualitative natural resource management systems, which are underpinned by Indigenous knowledges and worlds (Gadgil, Berkes, and Folke 2021). Yet, research indicates a troubling pattern of communicative exclusion reflected in the ways in which co-management has been carried out with many Indigenous communities (see Tam 2018; Theriault 2017; Parlee, Sandlos, and Natcher 2018), fuelled by the persistent legacy of a bureaucratic conservation approach shaped by scientific management.

These communicative tensions point toward the need for a critical consideration of communication within natural resource management. Yet, there exists a paucity of geographic research on communication in co-management with Indigenous communities (Tam 2018). This absence is emphasized by the unfolding ontological turn since the 2000s across multiple geographies and disciplines (Blaser 2014; Woolgar and Lezaun 2015). From a communications perspective, there is an emerging understanding that communicative tensions in co-management conventionally attributed to intercultural deficits may instead be understood as deeply rooted in ontological conflict. Understanding modern ontology as premised on Western values, worldview, knowledge system, and ideologies (Blaser 2013), such communicative conflict reflects the incommensurability between this dominant paradigm and many Indigenous ontologies (Theriault 2017; White 2020; Watson and Huntington 2014).

This paper explores the communicative implications of the ontological turn within natural resource co-management with Indigenous communities. Note that while this paper refers to Indigenous peoples and communities, this is grounded in the specific and distinct examples showcased in this paper drawn from global examples, and does not assume a universal experience. I situate this review within the intersections of critical social theory and three key bodies of literatures: natural resource management, participation and communication, and space and place. I begin by reviewing the ways in which communication has shifted within natural resource management alongside the rise of co-management in the 1980s and the emergence of adaptive co-management and adaptive governance approaches in the early 2000s. Despite these advances in recognizing the importance of building relationship and trust, the literature reflects the persistence of communicative tensions in co-management, underpinned by onto-epistemic violence. This paper demonstrates that while increasing care and attention has been accorded to the role of communication, particularly with the emergence of adaptive approaches, communicative tensions rooted in ontological difference persist in co-management with Indigenous communities. Synthesizing the literature, I identify and discuss four salient and generative threads and bring these together as the proposed idea of ethical equivocation—a communicative approach toward beginning to talk across worlds as equals within co-management with Indigenous communities. Research informing this review paper was carried out under the relevant ethics approvals.

## Positionality statement

Research is necessarily and inevitably shaped by the researcher; as Kovach (2009, 7) notes, “we know what we know from where we stand.” My research lens is shaped by my identity as a Southeast Asian woman in the modern diaspora, where I grew up as a “third-culture kid” (Mayberry 2016) having emigrated from my home country to the United Kingdom as a child. Learning to navigate “structures of whiteness” (Ahmed 2014; Todd 2016, 13) since young as a racialized minority has attuned me to the nuances of communication through experiences of communicative tensions when talking across worlds, fueled by conflicting ontological positions (including worldview, values, philosophy, ideologies) and the colonial present (experienced as microaggressions, racism, stereotypes). As a researcher on Indigenous lands and newcomer to Canada, where I live and work on Treaty 7 lands, my positionality is also shaped by my ongoing personal and professional journey in truth and reconciliation.

## Communication in natural resource management

### Scientific management and communicative action theory

Communication is non-dialogical and often confrontational in conventional natural resource management (NRM), which is characterized by power hierarchies, bureaucracy that is slow to respond, and scientism (Armitage et al. 2009). Promulgating technocratic policies that fail to account for the socio-ecological complexities of a changing world, conventional NRM has often been (and continues to be) at the expense of the environment and local peoples (Armitage, de Loë, and Plummer 2012; Armitage et al. 2009). Within the institutional fabric of natural resource management, the enduring influence of scientific management continues to challenge meaningful communication in shared decision-making. Scientific management is premised on a standardized system based on centralized, authoritarian control informed by technical, disinterested experts (referring to experts without a stake, or interest, in the matters under discussion) and rational science, in a top-down bureaucratic power hierarchy. This approach has been immensely successful since its emergence around 1900 in shaping fields spanning engineering, political ideology, organizational management, and governance (Olson 2015; Brunner et al. 2005)

Despite significant advances in the field toward working more meaningfully with communities in the past few decades through evolving approaches such as adaptive co-management, adaptive governance, and collaborative environmental governance, communication in co-management is challenged by the echoes of scientific management in Habermas’ (1990) communicative action theory. This theory has shaped the communicative ideal of decision-making as argumentation grounded in universal rationalism, with communicative norms which privilege formality, expertise, institutional authority, and language. Under communicative action theory, truth is determined through the “force of the better argument” (Habermas 1990, 88), among interlocutors who are assumed to be equal, within formal and highly structured institutional spaces of deliberation (Pieraccini and Cardwell 2016; Tam 2006; Tuler and Webler 2006). Habermas’ influence endures: Healy (2009, 1644) notes that within environmental decision-making, communicative action is used to “commonly frame attempts to facilitate public participation in technical decision-making.” Berkes’ (2010) tripartite model of adaptive co-management centres communicative action as one of three key pillars. In Sweden, communicative action is used as a theoretical benchmark against which effectiveness in forestry management is measured, to identify areas of improvement for dialogue across actors (Hertog and Brogaard 2021). In Nunavut, Tam (2018) observes communicative rationality in public hearings led by the Nunavut Wildlife Management Board, the territorial institution responsible for managing and regulating wildlife.

It is understandable why policymakers within institutions underpinned by Western governance ideals continue to embrace communicative action, which promises rational decision-making yielding universally valid judgements, through an ordered, inclusive process where all are equal, with deliberative legitimacy grounded in institutional authority. Communicative action is often adopted because of its claims in reaching a common understanding and vision, through a reflexive, deliberative process that considers the disparate values and knowledges that necessarily underpin much co-management. Yet, as Healy (2009, 1644) observes, Habermas’ assertions of “fair, free, and open forms of debate and communication” belie its execution in practice, which privileges Western communicative practices of confrontational argumentation, and scientific and technical knowledge. Scholarly literature further illuminates the ethnocentrism, scientism, technocracy, and exclusion embedded in communicative action (Young 2018; Allen 2012; Gunaratne 2006; Kaufman 1999; Buchstein and Jorke 2012), yet this communicative approach continues to shape spaces of public participation and decision-making across the global theatre within natural resource management.

Scholars call for the decolonization of communication ethics in such spaces which reflect “Western Enlightenment notions of rationality, justice, and humanity” (Ishii 2009; Munshi, Broadfoot, and Tuhiwai Smith 2011, 119). Miike (2007), for example, identifies Western biases in communicative practices that assert independence and individual rights, promulgate self-interest and ego, and privilege both rhetoric and reason, with a focus on speaking instead of on listening. Miike (2007, 276) summarizes: “Communication is thus conceived in the West as a process in which we manage ourselves and manipulate others or environments to achieve our individual goals and material comfort.” The problem arises when decision-making affecting Indigenous peoples are carried out in such spaces—a not uncommon situation within co-management given the tenacious entrenchment of Western bureaucratic ideology within the structure of institutions, including co-management boards (Watt-Cloutier 2016; White 2020).

### Communication and co-management approaches

The 1980s saw greater attention to communication through the emergence of co-management in response to the confluence of three trends: calls for meaningful citizen participation, the inability of the state to manage complex environmental challenges, and the recognition of the importance of local and traditional knowledges in improving outcomes (Fischer 2006; Berkes 2009; de Loë and Kreutzwiser 2007). This rise in co-management was fueled by growing disillusionment with centralized governance, the rising popularity of participation, and the mounting failures of scientific management toward the turn of the millennium (Brunner et al. 2005; Berkes 2010).

Social movements for Indigenous self-determination have further driven the growth of co-management and co-governance of natural resources (Watson and Huntington 2014). For example, the first Institute of Public Governance established under the Nunavut Agreement—the largest land claim at the time, ratified in 1993 after decades of efforts since the 1970s—was the Nunavut Wildlife Management Board (NWMB) in 1994. Established to manage wildlife within the territory, NWMB is critically mandated to ensure a voice for communities within decision-making that affects them (Nunavut Tunngavik Incorporated 2004). Understandings in co-management have been further enriched by emerging scholarship illuminating the kincentrism and deep relationality embedding in harvesting for many Indigenous peoples, such as Todd's (2014) “fish pluralities” and Kimmerer's “honourable harvest” (2015).

Co-management is envisioned to enable flexible and democratic decision-making to address more complex social challenges, enhance management outcomes, and empower communities in the process. Co-management centralizes the “co” through participatory processes that seek to meaningfully engage those most affected, and legitimize decisions made for and implemented in communities (Döll and Romero-Lankao 2017; Robards and Lovecraft 2010). Communication in co-management is critical to shared decision-making, which is essential to addressing complex environmental challenges, and dependent on meaningful participation (Silka, McGreavy, and Hart 2020; Tam 2019). Done well, participation in co-management enhances social learning, builds useful networks, cements policy ownership, fosters confidence, and a sense of belonging, enhances community knowledge and understanding, results in meaningful policy management outcomes, and cultivates positive perceptions toward the idea of co-management (Fischer 2006; Lovecraft, Meek, and Eicken 2013; Zhang et al. 2023; Major, Smith, and Migliano 2018). Done poorly, participation risks being subverted into a control mechanism to silence voices for political gain, erodes trust in state institutions and public officials, deepens citizen disengagement, creates suspicion toward project proponents, entrenches existing social inequities, and fosters feelings of frustration and alienation (Cooke and Kothari 2001; Puley and Charles 2022; Tam 2018; Nunan and Cepić 2020).

Yet, within the scholarly literature, public participation is commonly understood as a formal, official process (K. P. Hunt, Paliewicz, and Endres 2020), with scant attention paid to the spatial complexities of communication (Tam 2015, 2018, 2019). In response, spatial scholars such as Tam (2015, 128) urge reflection on “whose time, whose space, and whose terms for communication” are used in public participation. As Whyte (2021, 74) succinctly notes, “it's unreasonable to expect Indigenous people to participate at forums or in discussions where things are already defined in a way that doesn’t allow Indigenous people to meaningfully bring the issues that matter to them to the table.” Communicative conflict in co-management with Indigenous communities is not a new challenge. Within wildlife management, Mahoney (1983) called upon biologists to fulfil responsibilities in developing humility and skills in communication and facilitation. More than a decade later, Weeks and Packard (1997) called upon regulators and scientists in natural resource management to acknowledge and respect the pluralist worldviews of affected actors, and to recognize the critical nature of communication skills in achieving positive outcomes.

Adaptive co-management and adaptive governance emerged in the literature by the early 2000s (Plummer et al. 2012), to better address uncertainty and complexity through a heuristic approach based on “learning-by-doing” (McNabb and Swenson 2022). Adaptive co-management and adaptive governance are broad terms with oftentimes confusing definitions. Yet, what is clear across these approaches is the heightened importance accorded to voice and participation of affected communities in shared decision-making (Hasselman 2017), with a renewed focus on communication and collaboration (Plummer et al. 2012). Communicative elements common across adaptive approaches include building trusting relationships, co-producing multiple knowledges to improve socio-ecological outcomes, resolving tensions, nurturing social capital, and building social learning networks across key actors, toward establishing shared commitment and understanding (Lebel, Grothmann, and Siebenhüner 2010; Plummer et al. 2012; Ansell and Gash 2008; Armitage, de Loë, and Plummer 2012).

“Step zero” was proposed by Chuenpagdee et al. (2013) as a foundational phase to kick-off co-management in a good way, through forging common understanding and consensus among affected actors and co-management partners (prior to any implementation). Step zero acknowledges the need to start in a good way, together, and to continuously and reflexively respond to changing situations. This echoes Whyte's (2021) insight that Indigenous concepts of consent tend to reflect working together from the very start, at the site of *possible* collaboration. Adaptive approaches also espouse an explicit awareness of power relations across actors (Armitage et al. 2009), where power determines whose voice is heard, what knowledge is valorized, what kind of policy outcomes are desirable, how outcomes are measured and monitored, and how burdens and benefits are distributed. In these ways, communication in adaptive co-management and governance carefully considers the spatiality of participation. Such considerations arise from the spatial turn^1^ in the 1990s in communication studies, which recognizes the inextricability of humans from the spaces within which they are emplaced, and the ways in which space is riven with power. The ways in which spaces are necessarily socially constructed thus ground a relational ontology and a spatial politics (Massey 2005).

Adaptive co-management is still evolving and unfolding—its success, while demonstrable (see Plummer et al. 2017) is not (yet) a foregone conclusion. Measuring success across the multifaceted dimensions and complex trade-offs in co-management is challenging (Clark and Joe-Strack 2017). Time, in particular, is problematic—under a capitalist ideology where time is money, the need for speed impoverishes the heuristic nature of co-management and its core tenet of building relationship (Berkes 2010). While top-down approaches intrinsic to conventional NRM are perceived to be more efficient, particularly to bureaucrats and managers fearful of, and unequipped for, the “messy and problematic realms of community relations and consultation” (Howitt et al. 2013, 129), such shortcuts and false legitimation can result in violence and failure (Chuenpagdee and Jentoft 2007). Negative experiences, such as participation which yields no meaningful difference, discourage future collaboration (Muñoz-Erickson et al. 2010). In Arctic Canada, building trust and respect across different ways of being and knowing has, as Armitage et al. (2012) observed, necessarily taken decades. Yet taking this time is crucial, particularly in places with a legacy of colonization (Berkes et al. 2010). Where situations necessitate immediate action, collaboration cannot be done well (Ansell and Gash 2008) and it is preferable not to attempt collaborative strategies at all (Bodin 2017). As Tynan (2021, 599) succinctly states, “you cannot demand a relationship, nor give it a deadline.”

## The ontological turn and the persistence of Indigenous brilliance

If the spatial turn illuminated the exclusionary shortcomings in Habermas’ communicative action, the ontological turn cements the call to redress these gaps as no less than a political imperative toward truth and reconciliation. Emerging at the turn of the millennium, the ontological turn has provoked new avenues of research across fields such as cultural anthropology, science and technology studies, and critical geography (Blaser 2014; Woolgar and Lezaun 2015). While it is beyond the scope of this paper to map these avenues of research, research under the ontological turn has been varied and diverse, unsettling the dominant paradigm of modern ontology premised on Western values, worldview, knowledge system, and ideologies (Blaser 2013). Blaser (2014) highlights two (of many) different threads emergent under the ontological turn—within geography, a focus on more-than-human agency and the decentering of humans (K. Anderson 2014; Castree and Nash 2006), and within ethnography, a revitalized exploration of radical alterity that embraces multiple natures across different worlds in place of a single world, or nature, with multiple cultures (Blaser 2016; Viveiros de Castro 2015).

Significantly, the ontological turn reiterates that which Indigenous knowledges, peoples, and scholars have long elucidated, yet across much scholarship on the ontological turn, as Todd (2016, 6) observes, there is seldom attribution to the clear influence of “Indigenous thinkers for their millennia of engagement with sentient environments, with cosmologies that enmesh people into complex relationships between themselves and all relations.” Dispossession through “firsting”—described by Anderson and Christen (2019, 131) as the assertion of being the first to name, and therefore to claim, where “attribution is a site of ongoing settler-colonial power”—is reproduced in the erasure of Indigenous brilliance in Western academia (S. Hunt 2014; Simpson 2017). Yet, despite such erasure, Indigenous brilliance persists.

As Todd (2016) highlights, aspects of the ontological turn and its threads of more-than-human agency are echoed in many Indigenous worldviews. Sharing the Nuu-chah-nulth worldview, Atleo (2011, ix) discusses “tsawalk” (translated as “one”) as grounded within interdependent relationships, which unifies all “social, political, economic, constitutional, environmental, and philosophical issues … across all dimensions of reality—the material and the nonmaterial, the visible and the invisible.” Relationality as reality accords agency to relationships—imbued with a sense of responsibility and accountability—to create and shape that reality. As Tynan (2021, 600) states, “A relational reality … is an affective force that compels us to not just understand the world as relational, but feel the world as kin.”

Revelation, as Deloria (2003, 66) explains, is grounded in sacred landscapes; it is experiential, not abstract—“it was not what people believed to be true that was important but what they experienced as true.” Watts (2013) describes the concept of “Place-Thought” as the inextricable co-imbrication of place and thought, where “multiple worlds are indicative of each cosmology as being universally held to local places” (Watts 2016, 153). Place-thought is echoed in Simpson's (2017) seminal concept of “land as pedagogy,” where Indigenous intelligence is encoded in both the land and the complex practices of being on the land. It is also echoed in Coulthard's (2014, 13) concept of “grounded normativity,” which refers to governance that is grounded in place-based, relational and experiential thought and experience. Relationality across the land, humans, and non-humans (or more-than-humans) in these cosmological concepts reflects what Tynan (2021, 603) observes as “a relationalist ethos,” which “considers truths to be multiple and the land as the source of all meaning.”

These concepts are premised on the indivisibility of how we come to understand (epistemology) what we believe we understand (ontology) (Watts 2013). This indivisibility, reflected in the production of knowledge “in relationship to others” (Kovach 2009, 14), and as “self-in-relation” (Graveline 1998, 52), confronts the Western binary of epistemology and ontology premised on abstraction decoupled from place. Watts (2013, 28) cautions that dualisms, such as place/thought, humans/non-humans, ontology/epistemology, foster “spaces for colonial practices to occur.” Yet, such dualism, grounded in modern ontology and reflected in Habermas’ communicative norms, continues to shape spaces of participation for many Indigenous communities. Viveiros de Castro (2015, 9) characterizes this state of affairs as a shadow “war of worlds” illuminated by the ontological turn, with violence carried out under the banner of modern ontology.

## Ethical space, relationality, and Nuu-chah-nulth communication

Rose (1999, 176) observes that “a critical feature of the system [of Western thought and action] is that the ‘other’ never gets to talk back on its own terms.” This exclusionary state of affairs, shaped by the hegemony of modern ontology, is not for a lack of ontologically pluralist models of communication and engagement, which Indigenous critical scholars have discussed and conceptualized. Examples include Donald's (2012) ethical relationality, Ermine's (2007) ethical space, and Atleo's (2011) discussion of the communicative approach practiced by the Nuu-chah-nulth peoples. Atleo (2011, 46) describes the decision-making process of the ancient Nuu-chah-nulth as premised on three rights accorded to each person—the right to speak, to be heard, and to be understood, where “there was no debate, no argument, no interruptions, no point-counterpoint, but rather a process whereby each person in council exercised these three simple rules.” This approach accorded space for difference and honoured the Nuu-chah-nulth principles of recognition, consent, and continuity, imperative to harmony and balance within and across relations. Echoing practices grounded in Inuit ontology of abrogating the arrogance that one knows better (Qitsualik 2013), the Nuu-chah-nulth worldview highlights the importance of cultivating an “intentional state of humility” away from “self-serving egotism” in order to see others clearly (Atleo 2011, 85).

Reflecting such humility, ethical relationality decentres humans as part of a panoply of beings that coexist in the world as relations, and seeks to create bridges across difference through a deep understanding of self and other (Donald 2012). Drawing from the philosophies of the Plains Cree and Blackfoot, Donald (2012) proposes ethical relationality as a way to confront tensions across difference, without the confrontational need to capitulate or conquer. This approach is grounded in an attention and appreciation of the situatedness and emplacement of persons within a lively, relational world. Relatedly, Ermine (2007) adapts Poole's (1972) concept of ethical space to refer to the imagined, intersubjective reality created when societies with diverse worldviews intersect. Such a reality embraces different ways of knowing towards collaboration based on trusting relationships (Indigenous Circle of Experts 2018). Ethical space is a “refuge of possibility” towards a moral dialogue and partnership across worlds (Ermine 2007, 203). Dialogue unfolds under the “Indigenous gaze,” looking with one eye reflecting past injustice, and the other towards the future for their children.

Focusing on communication under the ontological turn “means to learn to be able to speak well” across ontological difference (Viveiros de Castro 2015, 14). Yet, communicative approaches such as ethical relationality, ethical space, and Nuu-chah-nulth learnings are rarely reflected in spaces of participation and decision-making. More common are the ways in which technologies of onto-epistemic violence are reflected within communication in co-management with Indigenous communities, as I highlight in the next section.

## Intercultural miscommunication, or ontological conflict?

Onto-epistemic violence in co-management communication manifests in myriad forms. Perhaps a suitable starting point is to explore the contention that exists in even naming this problem—intercultural miscommunication, or ontological conflict? Within co-management with Indigenous communities, communicative conflict that arises is commonly classified as intercultural misunderstanding, with resulting calls to improve intercultural communication, redress an “intercultural capacity deficit” (Howitt et al. 2013; Armitage, de Loë, and Plummer 2012), and for regulatory scientists to undergo cultural training (Watson 2013). Within public deliberation, Sass and Dryzek (2014, 8) observe that institutional spaces of decision-making and associated practices “are routinely treated as culture-free zones.” Yet for some, “culture is negotiable whereas the environment is not” (Blaser 2009, 15). When culture is viewed as one of many perspectives on a singular nature—knowable only by Western science—this embeds and entrenches ontological subjugation through practices of trivialization (Blaser 2014). Under modern ontology, Indigenous worlds and their ways of knowing and being are dismissed (and commonly derided) as belief, relegated to epistemology (de la Cadena 2010; Qitsualik 2013; Howitt et al. 2013). Presented as just a matter of perspective, cultures—and the worlds they encompass—are thus discounted.

Marisol de la Cadena (2010, 245) decries the hegemony of Western mononaturalism (one nature, many cultures) as a discriminatory “epistemic maneuver” that cannot be redressed through calls for multiculturalism. Blaser (2016) reiterates that naming conflicts as ontological, not cultural, begins to redress the trivialization of Indigenous peoples’ worlds as existing only epistemologically, by according ontological significance to Indigenous lands and lifeworlds. This classificatory change thus marks a seminal shift in thought that reinstates Fabian's (1983) “coevalness”—or equivalence—across diverse societies existing in “radical contemporaneity.” Importantly, the ontological turn is not simply a nomenclatural substitution of culture for ontology (although some contend it as such, see Carrithers et al. 2010)—it is a perspectival shift from a position of “ethnographic *authority*” to one of “ethnographic *alterity*” (Viveiros de Castro 2015, 4). Such a shift unsettles ontological superiority that seeks to subjugate and subordinate (Blaser 2009). To use the appropriate nomenclature is thus to begin to recognize the multiplexity of worlds, and to build bridges between them.

## Onto-epistemic violence in communication

Communication in co-management with Indigenous communities is commonly fraught with apprehension and anxiety, and marked by adverse and awkward interactions. For example, in northern Canada, Indigenous subsistence harvesters have been, and continue to be, unduly vilified as dismissive of land and ecological stewardship (Loo 2017; Parlee, Sandlos, and Natcher 2018). In Bolivia, conflict arose in the Pilón Lajas region, declared both a UNESCO Biosphere Reserve and Indigenous Territory, over disparate understandings of a lake—proposed as a site for tourism by the park authorities, but held as a spiritual, sacred place by the Mosetene people. Here, Gambon and Rist (2019, 61) conclude that this “lack of acceptance of simultaneously existing worlds, or ontologies” serves as “the biggest obstacle to a real dialogue.” In Alaska, Mitra (2018, 414) finds that Indigenous peoples were commonly excluded from workshops and roundtables on natural resource management, their otherness used as a rationale for being “*too* different and incapable of being engaged with.” At the heart of these tensions is a fundamental difference in how we see the world. As Gambon and Rist (2019) state, “Worldview matters.” Whyte et al. (2016) highlight that ontological assumptions, or worldviews, ground different approaches to the natural world. Relationality necessitates a humility and sense of responsibility to the natural world as intimate kin and kith, where such responsibility is fulfilled through land stewardship and reciprocity. Utilitarian approaches, in contrast, see through the lens of extractable resources and instrumentalize “resource inventories, wildlife assessments, [and] management programs” as mechanisms to quantify, control, and domesticate the environment (Natcher, Davis and Hickey 2005, 240).

Within co-management, there are calls for institutions and their staff to develop “new ethical competencies” that recognize ontological difference, and to confront the “legacies and contemporary manifestations of racialized, colonial discourses in many forms” (Howitt et al. 2013, 130). Siila (Sheila) Watt-Cloutier (2016, 318), Inuit climate leader, Nobel Peace Prize nominee and acclaimed author, observes “a lack of culture match between the institutions now in place in Inuit communities and our values and traditions.” White (2006) reiterates this challenge as “cultures in collision,” and identifies the Euro-Canadian values underpinning the Western bureaucratic system of co-management boards in northern Canada, which run counter to many Indigenous traditions, norms, and values. Among some Indigenous communities in northern Canada, communicative tensions are exacerbated by the practices of formality, bureaucracy, and legality among many co-management boards (White 2020). Yet, these cultural conflicts are deeply ontological in nature. Feelings of alienation in spaces of decision-making were reflected in Tam's (2018, 334) observations of caribou public hearings in western Nunavut, where Inuit participants shared the sentiment that “this hearing is not the Inuit way, but this is now the Nunavut way.” This echoes what Howitt et al. (2013, 135) refer to as the “technology of erasure,” where aspects of Indigenous governance are perceived as “unruly, intangible, illegible, and often invisible elements” that are ignored in formal processes and dismissed as “chaos and commotion.”

## Systems of legibility

Such dismissal reflects the colonizing and disenfranchising effects that institutional and state structure and processes can result in. Scholars have long highlighted that co-management with, or through, institutions shaped by Western governance norms, risks reproducing and entrenching hegemonic power and colonial relations. This enmeshes Indigenous peoples in processes and practices that serve to colonize (Howitt et al. 2013; Blaser 2009). In northern Canada, Walter Bayha (2012, 29), a member of the Dene Nation and previous Chair of the Sahtu Renewable Resources Board (SRRB), describes how he sought to reshape wildlife management to enshrine traditional ways, but hit an ontological brick wall:

… the Dene culture, their whole system, their worldview is different. It doesn’t work the same way as the federal and territorial legal systems. Their laws are different. Imagine trying to take a set of laws, like even as simple as wastage, the same way that Dene people think about it, and stick it into the Wildlife Act. It won’t work. We tried it. The federal/territorial legal systems don’t allow for the existence of protocols that don’t fit. The lawyers would say no, we can’t do that.

Pressure to conform is exerted by lawyers upholding hegemonic norms, as well as biologists and bureaucrats upholding Western approaches as epistemologically superior (Bayha 2012; Blaser 2009). This coercive conformity translates to an attendant need to be legible to dominant epistemology and ontology. This need to be legible is observed in the co-management of white-fronted geese with the Koyukon Athabascans in Alaska, United States. Watson (2013, 1097) describes the intimate and relational scale of traditional Koyukon knowledges which seek to develop instincts to “think like a goose.” This contrasts (and conflicts) deeply with the scientific credo of nonanthropomorphizing and the totalizing scale of scientific seeing—not the individual, but the group; not the animal, but its distinguishing characteristic. Legibility connotes power, where, as Watson (2013, 1098) notes, “Natives’ lack of political power comes not from whether they are at the management table, but from whether their different ontological and epistemological assumptions about nature and society produce information that is legible to the management regime.” In the Philippines, this need to be legible is echoed in environmental governance involving the Indigenous Palawan people, where recognition accorded to traditional knowledges is contingent upon “the expectation that Indigenous values and practices will work in harmony with bureaucratically managed conservation enclosures” (Theriault 2017, 115). Such “systems of legibility” thus ensure that while Indigeneity may be legally recognized, its subjugation by environmental regulation and policy serves to avoid the inconveniences of dealing with radical alterity.

Through these communicative contingencies of legibility within “structures of whiteness” (Ahmed 2014; Todd 2016, 13), modern ontology establishes firm limits on knowledge production and what counts as legitimate knowledge (Hunt 2014). Navigating such systems of legibility creates what Spivak describes as the forced homogenization of the “colonized body politic” through repressing vibrant heterogeneity and assimilating otherness (Darder and Griffiths 2018, 84). Within these systems, Indigenous teachings undergo transmutation into assimilated understandings, intangibly yet utterly different to their original meanings (Reid and Sieber 2020).

## Shapeshifting/living a dual life

For many Indigenous peoples, navigating systems of legibility necessitates what Hunt (2014, 28) describes as the critical skill of “shapeshifting, of moving between worlds.” For individuals, having to operate within these “structures of whiteness” (Ahmed 2014; Todd 2016, 13) may result in a deep inner conflict. Working in Western wildlife management systems often necessitates doing things in ways that are antithetical to many Indigenous teachings, risking colonization of, and violence to, the lifeworld. Bayha (2012) describes having to live a “dual life” during his time as a wildlife officer in the Northwest Territories. Compelled to carry out wildlife management activities that ran counter to his traditional teachings, Bayha (2012, 26) reflects how it was only upon his resignation that he was able to begin his “journey to become a human being, a true Dene.”

Within natural resource management, the need to shapeshift is also influenced by the dominant discourse in capacity building, which is oriented to remaking Indigenous peoples in compliance with the criteria and agenda of bureaucratic-scientific management (Howitt and Suchet-Pearson 2006; Howitt et al. 2013). Within spaces of decision-making and public participation, shapeshifting serves as a way to become legible to Habermas’ communicative norms. Yet, in shapeshifting and living dual lives, onto-epistemic violence is inflicted when people risk alienation from, or losing, their true selves, behind the many masks they are compelled to wear.

## Discussion: the idea of ethical equivocation

If we are to take the communicative implications of the ontological turn seriously, spaces of decision-making must begin to valorize communicative practices that fall outside the privileged realm of words and language. Further, these spaces must recognize that, as spatial scholars contend (Tam 2018; Foucault 1987; Bourdieu 1977), contrary to Habermas’ assumed equality across interlocutors, communication cannot be free of power. The idea of ethical equivocation is proposed here as bringing together four salient threads, discussed below, synthesized from this review across the intersections of critical social theory, natural resource management, participation and communication, and space and place. Ethical equivocation is presented here as a starting point toward thinking about the communicative consequences of the ontological turn in spaces of public participation and decision-making.

### Valorizing embodied and emplaced practices

First, it is important to carefully attend to and valorize embodied and emplaced practices as valid communication, beyond Habermas’ privileging of words, language, and speaking up. For many Indigenous peoples, governance and legal orders are grounded in everyday practices that guide ways of being and knowing, towards learning to live in a good way (Napoleon 2013; Mills 2016). As Hunt (2014, 31) explains, learning to dance at a Kwakwaka’wakw potlatch means learning “what it is to perform our law, our business, our spiritual obligations and relationships.” Ontologies are performed in these everyday practices, where worlds are not waiting to be discovered but are created through social practice (Law and Lien 2013; Mol 2002, 1999). Recalling Massey's (2005) insight that practice is the means by which we understand and shape the world and the spaces we exist in, we understand then that place is shaped by prescribed and proscribed behaviors (Seamon 2015; Cresswell 2015), where these social practices serve to inscribe and reinforce dominant ideologies (Althusser 2006). This raises the question—what ideologies, and whose worlds, are being (re)created in these spaces? Valorizing embodied and emplaced practices as communication—not just words—thus begins to unsettle the hegemony of modern ontology in these spaces of decision-making.

### Recognizing the impact of systemic colonialism on voice

Second, acknowledging colonization as a persistent and present structure of ongoing invasion on Indigenous lands, and not as an historical event (Wolfe 2006) more clearly unveils the deep roots of “colonial frontier logics” in discursive spaces that continue to parametrize participation and constrain communication for Indigenous peoples (Donald 2012, 533; Munshi, Broadfoot, and Tuhiwai Smith 2011). Systems of legibility, shaped by modern ontology, further reinforce power and colonial relations. Coulthard (2014) observes that within Canada, colonial power no longer manifests primarily through coercive means, but has become systemic. This is reflected in the uneven and mediated state recognition of, and limited accommodations for, Indigenous peoples. Far from the prevalent historicization of colonialism by “allocating the abuses of settler colonization to the dustbins of history” (108), Coulthard (2014, 14) contends that:

… authentic decolonization … has to account for the multifarious ways in which capitalism, patriarchy, white supremacy, and the totalizing character of state power interact with one another to form the constellation of power relations that sustain colonial patterns of behavior, structures, and relationships.

As illustrated in the phenomena of shapeshifting and living a dual life, ontological tensions are grounded in the degrees of incommensurability between Western and Indigenous worlds. Yet, the nuanced complexities in the impact of these tensions on voice and ability to speak iserased in spaces of decision-making that adopt Habermas’ assumption that all are equal (with equal ability to speak up).

### Caring for equivocations

Third, understanding that even the same word may hold disparate conceptual meanings across different worlds helps to ascertain that all parties share a common understanding. In Paraguay, Blaser (2009) describes the way in which biologists and bureaucrats understood sustainability through the lens of human–animal relations, in the form of hunting restrictions. In contrast, for the Indigenous Yshiro people, sustainability was secured through human–human relations. Their harvests were honoured through demonstrating respect and reciprocity in an ethics of care among the community. This is echoed in Inuit traditional practices of sharing country food harvests, particularly with those in need. These differences in what sustainability means are echoed in the different understandings behind the concept of respect. In northern Canada, Clark and Slocombe (2009) describe the ways in which respect for grizzly bears is demonstrated by Indigenous peoples through bear ceremonialism. This involves multifaceted practices such as storytelling to pass down customs and knowledge related to bears, addressing bears in kinship terms, enacting spiritual rituals, and embedding reciprocity in the bear–human relationship through staggering the use of salmon streams to share food resources. Respect for bears is thus reflected in these elements of a qualitative management approach centred on peaceful coexistence in a holistic ecosystem. This contrasts with practices of respect centred on ensuring a healthy population, through actions such as radio-collaring and extracting tissue samples from bears. Commonly justified in the name of the greater good, such actions appal many Indigenous peoples with their disrespect and harm to individuals regarded as more-than-human kin (Clark and Slocombe 2009).

Ontology thus shapes very different understandings behind seemingly shared words, particularly when it comes to concepts such as conservation, sustainability, and respect for nature. As Peterson et al. (2020, 238) state, “Notions of conservation are based on one's world view, and it cannot be assumed people will share the same concept, even within a given geographical area or cultural group.” Viveiros de Castro (2004, 2019) describes the process of parsing such disparate meanings for a generative way forwards as “controlling equivocations.” The concept of “equivocation” stands in contrast to “the univocal, as the claim to the existence of a unique and transcendent meaning” (Viveiros de Castro 2004, 11). Equivocations unfold in the liminal space when talking across worlds; controlling equivocations provoke scrutiny of the worlds brought into being by each speaker. Going further and drawing on Mol's (2008) definition of care, which connotes not control but deep attention to specificity and situatedness, Yates-Doerr (2019) suggests instead to care for, not control, equivocations. Caring for equivocations occurs through attention to context, positionality, and the ways in which relations change at the site of collaboration. In this way, caring for equivocations begins to foster a common understanding towards learning to talk across worlds.

### Becoming a good relation

Last, the idea of ethical equivocation is grounded in what I describe as the practice of ontography toward “ontographies of care.” Suspending ones’ own ontological assumptions begins to allow us to juxtapose and bridge our worlds (Viveiros de Castro 2015) by carefully feeling our way across. As Holbraad (2009, 90) succinctly puts it, “Alterity, in this sense, implies that we must always begin analysis in the dark, mired in misunderstanding.” Lynch (2013) defines ontography as the act of understanding, illustrating, and detailing the specific practices that comprise different worlds explored within the ontological turn. Rattasepp (2022, 365) further describes ontography as the process of mapping our relation to others, creating “ontographic descriptions as meeting points.” Ontography, as Viveiros de Castro (2013) and other scholars as discussed above describe, thus involves the imagining and mapping of different worlds, toward experiencing a world as others might experience it.

Yet, such intersubjective imagining may only be meaningfully carried out by those with a commitment to “a relational form of moral self,” which Munshi et al. (2011, 125) describe as an integral part of their call for a new communication ethics. This commitment is echoed in Inuit philosophies as exemplified in the concept of *Inummarik*—the Inuit ideal of a person that has attained sovereignty over self (Qitsualik 2013). With crucial characteristics of self-mastery, equanimity, patience, and a keen sense of relational interdependence (Snow and Obed 2022; Qitsualik 2013), *Inummarik* provides valuable learnings for engaging in encounter across worlds. From within the dominant paradigms of settler colonialism and capitalism, it is this messy, hard, and seemingly unending work of decolonizing the self (unlike the bounded parameters of ticking off a checklist or completing a training module) towards these concepts of *Inummarik* and Munshi et al.'s (2011, 125) “relational form of moral self” that renews relational responsibility and accountability, and revitalizes relational interdependence.

I suggest then, that ontography may also be understood as a practice—that of *becoming a good relation*, through learning to walk across worlds along the parallel paths of not only imagining different worlds, but also engaging in the deep work of decolonizing the self. Walking these iterative, nonlinear paths (that wind across the emic and etic) brings one closer to what Rose (1999, 184) describes as the “dialogical ethic of situated availability,” which is grounded in the principle of settlers situating human and non-human others first. Choosing vulnerability restores a mutuality that defines well-being as contingent upon an enmeshed interdependence in a relational web of shared subjectivities. As Rose (1999, 185) poignantly states: “We make ourselves vulnerable when we situate others first, whether those others are human or nonhuman, because we enter a world of mutuality in which there is no category of those who are exempt from suffering.”

Ontography, then, is not only a process, but the ongoing practice of becoming a good relation. Yet, as Indigenous and spatial scholars teach, place is practice, and practice is place. Walking these parallel paths brings into being what Harvey (2014), building on Foucault's ephemeral “spaces of difference” or heterotopias, describes as “spaces of hope” that hold the potential for other ways of being. Ontography, then, is also a place—a meeting place in the liminal, shifting spaces bridging different worlds. Doreen Massey (2004), a key theorist in the spatial turn, conceptualized “geographies of responsibility” to refer to the interweavings between place and person, and the relational interconnections that bind them. Moving beyond the spatial into the ontological turn, we might begin to extend this spatial concept of “geographies of responsibility” into the ontological dimension, toward what I describe as “ontographies of care” that begin to bridge different worlds.

## Conclusion

Communicative tensions occur when worlds collide. Onto-epistemic violence follows when spaces of participation and terms of engagement are determined in ways shaped by the dominant paradigm of modern ontology. This state of affairs reflects what Rose (1999, 184) describes as “deep colonizing,” where “practices of colonization are so institutionalized in political and bureaucratic structures and policies that they are almost unnoticed.” Howitt and Suchet-Pearson (2006) observe that such deep colonization risks compromising even imagination, where the hegemony of modern ontology acts to “place limits upon alterity and therefore … upon thought also” (Holbraad, Pedersen, and Viveiros de Castro 2014). Yet, as Berkes and Nayak (2018) conclude, for meaningful co-management by and with communities, where healthy communities mean healthy environments, “one would first need to imagine it.” A fundamental rethinking is necessary—“it is not just the relationships of power that need to be reshaped, but also the concepts, language, and images used to describe, analyze and address the processes” (Howitt and Suchet-Pearson 2006, 324).

Rethinking communication under the ontological turn in co-management with Indigenous communities, the idea of ethical equivocation brings together four salient threads from the literature. Ethical equivocation is presented as a starting point towards rethinking communication (particularly within systems of legibility) through its use as a communicative tool to be adapted to local specificities, contexts, and needs by those involved in co-management with Indigenous communities. Specifically, ethical equivocation calls upon political and co-management institutions, as well as non-Indigenous persons involved in decision-making that affects Indigenous peoples, to foster pluralist spaces of public participation and decision-making that facilitate talking across worlds. As a starting point, ethical equivocation calls for these institutions and individuals to valorize embodied and emplaced practices as valid communication, recognize the impact of systemic colonialism on voice, care for equivocations, and to deeply engage in the ongoing practice of becoming a good relation.

This approach of ethical equivocation does not exist in a vacuum—it draws on the spatial turn, is situated within the unfolding ontological turn, and is influenced by communicative approaches espoused by Indigenous critical scholars such as Donald's (2012) ethical relationality and Ermine's (2007) adaptation of Poole's (1972) concept of ethical space. Unfolding transformations as part of Indigenous resurgence include the reinvigoration of governance structures, systems, and institutions around a relational ethos and ecological kincentrism (Whyte 2021; Simpson 2017). The idea of ethical equivocation—proposed here as a communicative tool within co-management with Indigenous communities—seeks to contribute toward the ongoing conversation, among scholars and practitioners, in learning to talk across worlds. More broadly, this paper synthesizes the scholarly literature in the hopes that this communicative tool might assist in bridging the gap between theory and practice in co-management communication. Talking across worlds is becoming increasingly critical in the face of climate change and growing environmental pressures, which not only compound the challenging nature of co-management, but also disproportionately and inequitably adversely impact many Indigenous communities. Globally, ongoing struggles for resources on Indigenous lands continue to ensue in violent conflict and entrench environmental injustice (Mamo 2024). Here, the encompassing ontological understandings of “land” as holistic landscapes (indivisible by imaginaries of borders and barriers) for many Indigenous peoples also drive communicative conflict (Whyte 2021). Recognizing, refuting, and remediating onto-epistemic violence in spaces of public participation and decision-making is a step toward overcoming communicative exclusion in co-management, toward learning to talk across worlds.

## Notes

1. The spatial turn vitalizes Fabian's (1983) premise of “radical contemporaneity,” which regards diverse societal configurations and choices as equal and valid. The spatial turn is shaped by concepts such as Lefebvre's (1991) “spatial code” or language situated in the critical and reflexive knowledges that centre lived experience and embrace the unknown; Heidegger's concept of *dasein*, which illuminates an embodied way of being-in-the-world, or the spatiality of being; and Foucault's advocacy for returning to the vitality and vibrancy of space, understood as structured by social, cultural, and political relations (Soja 2009).
